# Enhanced mortality prediction in pneumonia-associated acute respiratory distress syndrome: a model integrating lymphocyte subsets with clinical parameters in non-immunosuppressed adults

**DOI:** 10.3389/fmed.2026.1844614

**Published:** 2026-05-20

**Authors:** Zhen-Chuan Xing, Hua-Zheng Guo, Ting Ao, Jin-Xiang Wang, Ming Hu

**Affiliations:** 1 Department of Pulmonary and Critical Care Medicine, Beijing Luhe Hospital, Capital Medical University, Beijing, China; 2 Department of Infectious Disease, Beijing Luhe Hospital, Capital Medical University, Beijing, China

**Keywords:** acute respiratory distress syndrome, CD4+ T cell count, mortality prediction, pneumonia, sequential organ failure assessment score

## Abstract

**Background:**

Acute respiratory distress syndrome (ARDS) is a common clinical syndrome among critically ill patients and is associated with high mortality. Although scoring tools based on organ dysfunction currently exist, their ability to assess the immune status of patients remains limited. This study aimed to develop a predictive model for in-hospital mortality in non-immunosuppressed adults with pneumonia-associated ARDS by integrating clinical and host immune parameters.

**Methods:**

This single-center retrospective study included 126 adults with p-ARDS. Data collected within 48 h of admission were analyzed. Candidate predictors (*p* < 0.10 in univariate analysis, excluding post-admission treatments) underwent simultaneous selection by BIC-based best subset selection and LASSO regression; variables identified by both methods were retained. A multivariable model combining selected predictors with the SOFA score (Model 2) was constructed, and a SOFA-only model served as the baseline (Model 1). Performance was evaluated for discrimination, calibration, and clinical utility. Bootstrap internal validation (500 iterations) was performed.

**Results:**

The overall in-hospital mortality rate was 35.7% (45/126). The final combined model incorporated the following variables: heart failure (aOR = 5.207), CD3 + CD4 + T-cell count (aOR = 0.719 per 100 cells/μL), D-dimer (aOR = 1.113 per 1 μg/mL) and SOFA score (aOR = 1.126 per point). The model demonstrated excellent predictive performance, with an AUC of 0.805 (95% CI: 0.730–0.879), which was significantly higher than that of the baseline model (AUC: 0.707; 95% CI: 0.618–0.797; *p* = 0.020). Calibration curves and decision curve analysis further confirmed its good calibration and superior clinical net benefit. Bootstrap internal validation demonstrated robustness (optimism-corrected C-statistic: 0.788).

**Conclusion:**

We developed a multidimensional model for predicting in-hospital mortality in non-immunosuppressed adult patients with p-ARDS. The model’s performance was superior to the SOFA score. External validation is needed for the widespread application of this model.

## 1 Introduction

Acute respiratory distress syndrome (ARDS) is a critical clinical syndrome characterized by diffuse pulmonary inflammation and damage to the alveolar epithelial-capillary barrier. The clinical, physiological, biological and imaging manifestations of ARDS are highly heterogeneous. It is primarily be divided into two types: intrapulmonary and extrapulmonary. The condition manifests as progressive respiratory distress and refractory hypoxemia (1–3). Despite substantial advancements in mechanical ventilation strategies and supportive care in recent years, ARDS continues to be a relatively frequent condition associated with high morbidity and mortality, with severe ARDS cases exhibiting mortality rates as high as 46–66% (4–7). Pneumonia constitutes a major etiological factor for ARDS, accounting for approximately 59% of all ARDS cases (5). In patients with pneumonia-associated acute respiratory distress syndrome (p-ARDS), the interplay between the initial pathogen invasion and the dysregulated host immune response serves as a critical determinant of clinical progression and prognosis.

The Sequential Organ Failure Assessment (SOFA) score and the CURB-65 score are clinically established tools for quantifying the extent of organ dysfunction and predicting mortality in pneumonia patients. These tools primarily serve to assess the extent of physiological dysfunction but demonstrate significant limitations in evaluating underlying pathophysiological mechanisms, particularly the immune status. In studies of COVID-19-related ARDS, immune dysregulation has been identified as a core feature of the disease pathophysiology, typically manifesting as significant lymphopenia and alterations in the distribution and function of key lymphocyte subsets, such as helper T cells (8). Meanwhile, pertinent studies have confirmed that adaptive immune impairment occurs during sepsis. The increased apoptosis of T cells, B cells, and dendritic cells drives a shift in the immune system from an initial pro-inflammatory state toward an anti-inflammatory and immunosuppressive response, potentially resulting in immunosuppression (9). Multiple retrospective studies have demonstrated that persistent lymphopenia in sepsis patients is associated with an elevated risk of mortality and a heightened incidence of hospital-acquired infections (10–12). These findings underscore the potential value of integrating quantifiable biomarkers that reflect the host immune status into established risk assessment frameworks. Such an approach may address the limitations of conventional scoring systems and allow for more precise prognostic stratification of patients with ARDS.

This study aims to investigate non-immunosuppressed adults with pneumonia-related ARDS by systematically analyzing clinical and laboratory factors associated with in-hospital mortality. By integrating easily accessible clinical indicators and biomarkers reflecting immune function, we constructed and validated a predictive model for in-hospital mortality risk specifically. Our objective was to facilitate precise prognostic stratification in this high-risk subgroup and provide an evidence-based foundation for future individualized management.

## 2 Materials and methods

### 2.1 Study population

**Figure 1:**
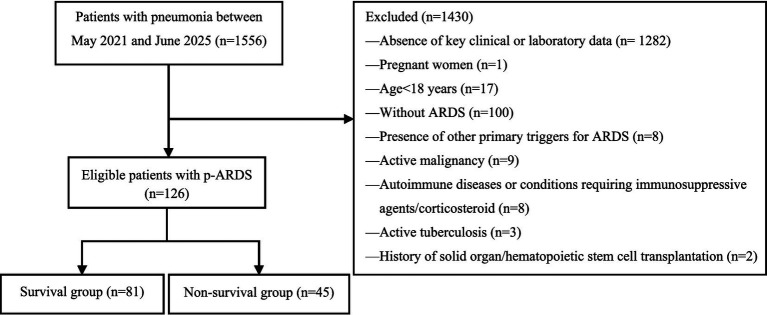
Flow chart of inclusion and exclusion criteria of participants.

This study was a single-center retrospective observational study. The study screened adult patients with pneumonia-induced acute respiratory distress syndrome (ARDS) who were admitted to the Department of Infectious Diseases, Beijing Luhe Hospital, Capital Medical University between May 2021 and June 2025. Inclusion criteria: (i) Age ≥ 18 years; (ii) Pneumonia diagnosis meeting the criteria of the American Thoracic Society/Infectious Diseases Society of America guidelines (13); (iii) ARDS diagnosis meeting the Berlin definition (14) and the new global definition of ARDS (3). Exclusion criteria: (i) Active malignancy; (ii) Autoimmune diseases or conditions requiring immunosuppressive agents or corticosteroid therapy; (iii) Active tuberculosis; (iv) History of solid organ or hematopoietic stem cell transplantation; (v) Presence of other primary triggers for ARDS (e.g., severe trauma, acute pancreatitis); (vi) Absence of key clinical or laboratory data. After the screening process, a total of 126 patients were included in the final analysis. Based on the in-hospital outcome, patients were categorized into the survival group (n = 81) and the non-survival group (n = 45) (Figure 1). This study aimed to identify independent risk factors for in-hospital mortality by comparing the clinical characteristics of the two groups. Subsequently, we developed and validated a predictive model for in-hospital mortality that incorporates both the SOFA score and the identified risk factors, evaluating its performance against a baseline model using the SOFA score alone. The study was approved by the ethics committee of Beijing Luhe Hospital, Capital Medical University (2025-LHKY-017-01). All patient data were collected from the hospital information system (HIS).

### 2.2 Data collection

The collected demographic and clinical data included: (1) age and sex; (2) underlying diseases: hypertension, ischemic heart disease, cerebrovascular disease, diabetes mellitus, chronic lung disease, chronic liver disease, chronic kidney disease, and heart failure; (3) laboratory parameters: peripheral blood white blood cell count (WBC), neutrophil count, lymphocyte count, platelet count (PLT), neutrophil-to-lymphocyte ratio (NLR), peripheral blood lymphocyte subsets, serum ferritin, procalcitonin (PCT), C-reactive protein (CRP), interleukin-6 (IL-6), albumin (ALB), alanine aminotransferase (ALT), aspartate aminotransferase (AST), total bilirubin (Tbil), serum creatinine, urea, creatine kinase (CK), lactate dehydrogenase (LDH), prothrombin time (PT), activated partial thromboplastin time (APTT), thrombin time (TT), fibrinogen, D-dimer, and PaO₂/FiO₂ ratio; (5) pathogens; (6) SOFA score and CURB-65 score; (7) use of vasoactive drugs, continuous renal replacement therapy (CRRT), mechanical ventilation, length of hospital stay, and in-hospital mortality. All laboratory tests, scoring assessments, and ARDS diagnosis were completed within 48 h of admission.

### 2.3 Statistical analysis

Continuous variables with a normal distribution were expressed as mean ± standard deviation and compared using the independent samples t-test. Non-normally distributed variables were summarized as median (interquartile range) and analyzed using the Mann–Whitney U test. Categorical variables were presented as number (percentage) and compared with the chi-square test or Fisher’s exact test, as appropriate. Complete case analysis was used without imputation for missing values. The proportion of missingness for the primary predictor, lymphocyte subsets, was extremely high (>80%) and followed a non-random missing mechanism. Imputation methods could introduce unreliable estimates; therefore, only the 126 patients with complete data were included in the final analysis.

Univariate logistic regression was first performed to identify factors associated with in-hospital mortality. Variables with a *p* < 0.10 in univariate analysis, except post-admission treatment modalities (which were excluded as they represent post-admission interventions), were included in the subsequent variable selection process. To mitigate the limitations of a single variable selection method, two approaches were applied: (1) best subset selection based on the Bayesian Information Criterion (BIC); and (2) LASSO regression with family = binomial, where the optimal tuning parameter *λ* was selected via five-fold cross-validation using the one-standard-error rule to determine λ.1se. Variables jointly identified by both methods were retained. Multicollinearity among these variables was diagnosed using the variance inflation factor (VIF); any variable with VIF > 5 was removed.

A multivariable logistic regression model was constructed using the selected predictors combined with the SOFA score. To compare the predictive model developed in this study (Model 2) with the baseline model consisting solely of the SOFA score (Model 1), model performance was evaluated from three aspects: discrimination, calibration, and clinical utility. Discrimination was assessed using the receiver operating characteristic (ROC) curve and the area under the curve (AUC), and the DeLong test was applied to compare the AUCs between the two models. Calibration was evaluated using the Hosmer-Lemeshow goodness-of-fit test, a calibration plot, and the Brier score. Clinical utility was assessed using decision curve analysis to evaluate the net benefit at different threshold probabilities. To correct for optimism bias potentially resulting from model overfitting, internal validation of the final model (Model 2) was performed using bootstrap resampling with 500 iterations. The optimism-corrected AUC was calculated. Statistical analyses were performed using Free Statistics analysis platform (Version 2.0, Beijing, China). Statistical significance was set at a two-sided *p* < 0.05 for final model coefficients and primary comparisons. This study adheres to the TRIPOD+AI (Transparent Reporting of a multivariable prediction model for Individual Prognosis Or Diagnosis + Artificial Intelligence, 2024 version) reporting guideline.

## 3 Results

### 3.1 Clinical characteristics

A total of 126 patients were enrolled in this study with a mean age of 74.0 ± 10.4 years (range: 49–98), and the majority were male (70.6%, *n* = 89). Comorbidities were revalent, particularly hypertension (62.7%), heart failure (56.3%), cerebrovascular disease (40.5%), and diabetes mellitus (41.3%). Chronic lung disease, ischemic heart disease, and chronic kidney disease were present in 15.9, 17.5, and 18.3% of patients, respectively.

Laboratory investigations on admission revealed prominent inflammatory and organ dysfunction markers. Leukocytosis, elevated neutrophil count, lymphopenia, and markedly increased levels of CRP, PCT, IL-6, and serum ferritin were observed. Hypoalbuminemia and coagulation abnormalities, including prolonged prothrombin time, elevated fibrinogen, and increased D-dimer, were common. Immune profiling demonstrated significant depletion of T-cell subsets (CD3^+^, CD3^+^CD4^+^, CD3^+^CD8^+^), natural killer (NK) cells, and B cells.

The pathogen profile revealed infection rates of 33.3% for SARS-CoV-2, 5.6% for influenza virus, and 6.3% for Aspergillus. The distribution of ARDS severity among the 126 patients was mild in 54 (42.9%), moderate in 54 (42.9%), and severe in 18 (14.3%), according to the Berlin definition. The cohort had a median SOFA score of 7.4 ± 3.6 points. According to the CURB-65 criteria for pneumonia severity, 56.3% (71/126) of patients were classified as high risk (score ≥3). The median length of hospital stay was 14.0 (9.0, 20.8) days. During hospitalization, 51.6% of patients received vasoactive medications, 12.7% underwent CRRT, and 45.2% required mechanical ventilation. The all-cause in-hospital mortality rate was 35.7% (n = 45) (shown in Table 1).

**Table 1:** Baseline characteristics.

Variables	Normal reference range	Total (*n* = 126)
Male, *n* (%)		89 (70.6)
Age, (years)		74.0 ± 10.4
Medical history		
Hypertension, *n* (%)		79 (62.7)
Ischemic heart disease, *n* (%)		22 (17.5)
Cerebrovascular disease, *n* (%)		51 (40.5)
Diabetes mellitus, *n* (%)		52 (41.3)
Chronic lung disease, *n* (%)		20 (15.9)
Chronic liver disease, *n* (%)		1 (0.8)
Chronic kidney disease, *n* (%)		23 (18.3)
Heart failure, *n* (%)		71 (56.3)
Laboratory investigations on admission		
White blood cell, (×10^9^/L)	3.5–9.5	9.7 ± 6.0
Neutrophil, (×10^9^/L)	1.8–6.3	7.3 (4.2, 11.0)
Lymphocyte, (×10^9^/L)	1.1–3.2	0.7 ± 0.4
NLR		12.1 (6.6, 22.6)
Platelet, (×10^9^/L)	125–350	189.5 ± 93.2
C-reactive protein, (mg/L)	<10	142.3 (61.6, 200.0)
Procalcitonin, (ng/mL)	<0.05	0.5 (0.1, 6.3)
Interleukin-6, (pg/mL)	<7	79.3 (24.3, 463.5)
Serum ferritin,(ng/mL)	4.63–204.00	674.6 (340.2, 1269.0)
Albumin, (g/L)	40–55	32.1 ± 4.5
ALT, (U/L)	0–40	21.5 (12.2, 41.0)
AST, (U/L)	0–35	29.0 (19.0, 46.8)
Total bilirubin, (μmol/L)	0–23	9.8 (6.5, 13.6)
Serum creatinine, (μmol/L)	41–73	83.5 (63.0, 155.8)
Urea, (mmol/L)	2.6–7.5	9.2 (6.1, 15.1)
Creatine kinase, (U/L)	40–200	100.5 (49.0, 273.0)
Lactate dehydrogenase, (U/L)	120–250	286.0 (202.2, 378.8)
Prothrombin time, (S)	9.4–12.5	13.2 ± 3.1
APTT, (S)	25.4–38.4	31.9 ± 6.4
Thrombin time, (S)	10.3–16.6	14.1 (13.2, 15.7)
Fibrinogen, (g/L)	2.00–4.00	5.2 ± 2.3
D-Dimer, (μg/mL)	0–0.243	0.9 (0.5, 2.2)
PaO₂/FiO₂ ratio, (mmHg)	>400	180.6 ± 69.9
Peripheral blood lymphocyte subsets		
T cells (CD3+)/μL	940–2,140	366.5 (239.2, 619.8)
T cells (CD3 + CD8+)/μL	380–790	133.0 (79.0, 251.5)
T cells(CD3 + CD4+)/μL	550–1,200	194.5 (121.2, 332.0)
NK cells (CD16 + CD56+)/μL	155–550	107.5 (56.5, 215.5)
B cells (CD19+)/μL	160–350	83.5 (47.0, 150.8)
CD4+/CD8+ ratio	0.9–2.0	1.5 (0.9, 2.4)
SARS-CoV-2, *n* (%)		42 (33.3)
Influenza, *n* (%)		7 (5.6)
Aspergillus, *n* (%)		8 (6.3)
ARDS Classification, *n* (%)		
Mild		54 (42.9)
Moderate		54 (42.9)
Severe		18 (14.3)
SOFA score, points		7.4 ± 3.6
CURB-65 score, *n* (%)		
<3		55 (43.7)
≥3		71 (56.3)
Vasoactive drugs, *n* (%)		65 (51.6)
CRRT, *n* (%)		16 (12.7)
Mechanical ventilation, *n* (%)		57 (45.2)
Length of stay (days)		14.0 (9.0, 20.8)
In-hospital mortality, *n* (%)		45 (35.7)

NLR, neutrophil-to-lymphocyte ratio; ALT, alanine aminotransferase; AST, aspartate aminotransferase; APTT, activated partial thromboplastin time; SOFA, Sequential Organ Failure Assessment; CRRT, continuous renal replacement therapy.

### 3.2 Factors associated with mortality in univariate analysis

No significant differences were found in age or gender distribution between the survivors group and the non-survivors group. Univariate analysis identified ischemic heart disease (OR = 2.582, 95%CI: 1.013–6.579, *p* = 0.047), chronic kidney disease (OR = 2.884, 95%CI: 1.145–7.267, *p* = 0.025), and heart failure (OR = 6.393, 95%CI: 2.645–15.452, *p* < 0.001) as significant risk factors for in-hospital mortality. In contrast, a history of hypertension, diabetes mellitus, or cerebrovascular disease showed no significant association (Table 2).

**Table 2:** Univariate analysis of risk factors associated with death events.

Variables	Survivors (*n* = 81)	Non-survivors (*n* = 45)	OR (95% CI)	*p*
Male, *n* (%)	57 (70.4)	32 (71.1)	1.036 (0.465–2.311)	0.930
Age, (years)	73.8 ± 11.3	74.4 ± 8.6	1.006 (0.971–1.042)	0.733
Medical history				
Hypertension, *n* (%)	49 (60.5)	30 (66.7)	1.306 (0.609–2.802)	0.493
Ischemic heart disease, *n* (%)	10 (12.3)	12 (26.7)	2.582 (1.013–6.579)	0.047
Cerebrovascular disease, *n* (%)	29 (35.8)	22 (48.9)	1.715 (0.818–3.596)	0.153
Diabetes mellitus, *n* (%)	34 (42.0)	18 (40.0)	0.922 (0.439–1.935)	0.829
Chronic lung disease, *n* (%)	12 (14.8)	8 (17.8)	1.243 (0.467–3.312)	0.663
Chronic liver disease, *n* (%)	1 (1.2)	0 (0.0)	–	0.987^*^
Chronic kidney disease, *n* (%)	10 (12.3)	13 (28.9)	2.884 (1.145–7.267)	0.025
Heart failure, *n* (%)	34 (42.0)	37 (82.2)	6.393 (2.645–15.452)	<0.001
Laboratory test on admission				
White blood cell, (×109/L)	9.3 ± 5.9	10.4 ± 6.2	1.030 (0.970–1.094)	0.331
Neutrophil, (×109/L)	6.9 (3.9, 10.5)	7.9 (5.2, 12.1)	1.039 (0.977–1.105)	0.227
Lymphocyte, (×109/L)	0.7 ± 0.4	0.6 ± 0.4	0.415 (0.153–1.131)	0.086
NLR	10.3 (5.8, 20.0)	13.7 (8.0, 29.6)	1.013 (1.000–1.027)	0.051
Platelet, (×109/L)	193.4 ± 95.5	182.4 ± 89.4	0.999 (0.995–1.003)	0.528
C-reactive protein, (mg/L)	119.9 (54.0, 200.0)	180.3 (96.7, 200.0)	1.001 (0.999–1.003)	0.464
Procalcitonin, (ng/mL)	0.3 (0.1, 2.8)	1.1 (0.3, 11.0)	1.014 (0.993–1.036)	0.187
Interleukin-6, (pg/mL)	57.0 (15.4, 256.7)	172.4 (39.3, 819.9)	1.000 (1.000–1.000)	0.527
Serum ferritin,(ng/mL)	596.3 (337.8, 1064.6)	909.6 (394.8, 1648.6)	1.000 (1.000–1.000)	0.081
Albumin, (g/L)	32.8 ± 4.1	30.8 ± 4.8	0.900 (0.824–0.982)	0.017
ALT, (U/L)	22.0 (12.0, 41.0)	20.0 (14.0, 37.0)	1.004 (0.998–1.011)	0.206
AST, (U/L)	27.0 (18.0, 44.0)	30.0 (19.0, 67.0)	1.002 (0.999–1.006)	0.191
Total bilirubin, (μmol/L)	9.6 (6.8, 13.2)	10.2 (6.4, 14.7)	1.012 (0.969–1.058)	0.589
Serum creatinine, (μmol/L)	80.0 (65.0, 121.0)	87.0 (62.0, 170.0)	1.001 (0.999–1.004)	0.196
Urea, (mmol/L)	9.1 (5.8, 14.7)	10.0 (6.4, 15.3)	1.010 (0.982–1.039)	0.495
Creatine kinase, (U/L)	90.0 (49.0, 246.0)	113.0 (49.0, 293.0)	1.000 (1.000–1.000)	0.571
LDH (U/L)	251.0 (193.0, 351.0)	340.0 (226.0, 529.0)	1.002 (1.000–1.004)	0.028
Prothrombin time, (S)	13.3 ± 3.0	13.0 ± 3.3	0.969 (0.859–1.093)	0.611
APTT, (S)	31.1 ± 6.6	33.2 ± 5.9	1.055 (0.994–1.118)	0.076
Thrombin time, (S)	14.0 (13.1, 15.2)	14.8 (13.3, 16.4)	1.083 (0.916–1.280)	0.352
Fibrinogen, (g/L)	5.2 ± 2.3	5.1 ± 2.3	0.994 (0.845–1.168)	0.939
D-Dimer, (μg/mL)	0.7 (0.4, 1.6)	1.3 (0.8, 2.6)	1.152 (0.977–1.358)	0.093
PaO₂/FiO₂ ratio, (mmHg)	194.7 ± 64.1	155.3 ± 73.2	0.992 (0.986–0.997)	0.003
T cells (CD3+)/μL	448.0 (265.0, 641.0)	279.0 (188.0, 505.0)	0.998 (0.997–1.000)	0.011
T cells (CD3 + CD8+)/μL	157.0 (93.0, 278.0)	105.0 (68.0, 170.0)	0.997 (0.995–1.000)	0.070
T cells (CD3 + CD4+)/μL	227.0 (150.0, 353.0)	141.0 (96.0, 214.0)	0.997 (0.994–0.999)	0.013
NK cells (CD16 + CD56+)/μL	118.0 (67.0, 232.0)	93.0 (48.0, 164.0)	0.999 (0.996–1.002)	0.390
B cells (CD19+)/μL	87.0 (52.0, 171.0)	74.0 (28.0, 148.0)	0.998 (0.994–1.001)	0.190
CD4+/CD8 + ratio	1.4 (0.9, 2.4)	1.6 (0.9, 2.4)	1.162 (0.918–1.468)	0.212
SARS-CoV-2, *n* (%)	23 (28.4)	19 (42.2)	1.843 (0.859–3.955)	0.117
Influenza, *n* (%)	7 (8.6)	0 (0.0)	–	0.986^*^
Aspergillus, *n* (%)	5 (6.2)	3 (6.7)	1.086 (0.247–4.770)	0.913
ARDS (moderate-to-severe) *n* (%)	42 (51.9)	30 (66.7)	1.857 (0.871–3.962)	0.109
SOFA score, points	6.6 ± 3.8	9.0 ± 2.4	1.220 (1.088–1.368)	0.001
CURB-65 score ≥3, *n* (%)	43 (53.1)	28 (62.2)	1.456 (0.692–3.063)	0.323
Vasoactive.drugs, *n* (%)	28 (34.6)	37 (82.2)	8.755 (3.592–21.338)	<0.001
CRRT, *n* (%)	3 (3.7)	13 (28.9)	10.563 (2.819–39.584)	<0.001
Mechanical ventilation, *n* (%)	22 (27.2)	35 (77.8)	9.386 (3.986–22.105)	<0.001
Length of stay (days)	15.0 (9.0, 22.0)	13.0 (5.0, 17.0)	0.963 (0.928–0.999)	0.043

NLR, neutrophil-to-lymphocyte ratio; ALT, alanine aminotransferase; AST, aspartate aminotransferase; APTT, activated partial thromboplastin time; SOFA, Sequential Organ Failure Assessment; CRRT, continuous renal replacement therapy; LDH, lactate dehydrogenase.

^*^Due to the small number of events, the OR and its 95% CI could not be reliably estimated.

Univariate analysis of laboratory tests at admission showed that the non-survivors group had significantly lower serum albumin levels (30.8 ± 4.8 g/L vs. 32.8 ± 4.1 g/L; OR = 0.900, 95%CI: 0.824–0.982, *p* = 0.017), PaO₂/FiO₂ ratio (155.3 ± 73.2 mmHg vs. 194.7 ± 64.1 mmHg; OR = 0.992, 95% CI: 0.986–0.997, *p* = 0.003), CD3 + T cell counts (279.0/μL vs. 448.0/μL; OR = 0.998, 95% CI: 0.997–1.000, *p* = 0.011) and CD3 + CD4 + T cell counts (141.0/μL vs. 227.0/μL; OR = 0.997, 95% CI: 0.994–0.999, *p* = 0.013). Meanwhile, lactate dehydrogenase levels were significantly higher in the non-survivors group (340.0 U/L vs. 251.0 U/L, *p* = 0.028). A trend toward a higher NLR was observed in non-survivors (median, 13.7 vs. 10.3), although this difference reached only borderline statistical significance (OR = 1.013, 95% CI: 1.000–1.027, *p* = 0.051). There were no statistically significant differences between the two groups in white blood cell count, CRP, PCT, and other infection and inflammation markers, as well as liver and kidney function indicators.

The SOFA score was significantly higher in the non-survivor group than in the survivor group (9.0 vs. 6.6 points; OR = 1.220, 95%CI: 1.088–1.368, *p* = 0.001), whereas there were no statistically significant differences between the two groups in the proportion of patients with CURB-65 ≥ 3 or in the proportion of patients with moderate-to-severe ARDS. Non-survivors received vasoactive drugs (OR = 8.755, 95% CI: 3.592–21.338, *p* < 0.001), CRRT (OR = 10.563, 95%CI: 2.819–39.584, *p* < 0.001), and mechanical ventilation (OR = 9.386, 95%CI: 3.986–22.105, *p* < 0.001) at significantly higher rates than survivors. No statistically significant differences were observed between two groups in the etiological distribution (SARS-CoV-2, influenza, and Aspergillus).

### 3.3 Development and performance of the multivariable model

**Figure 2:**
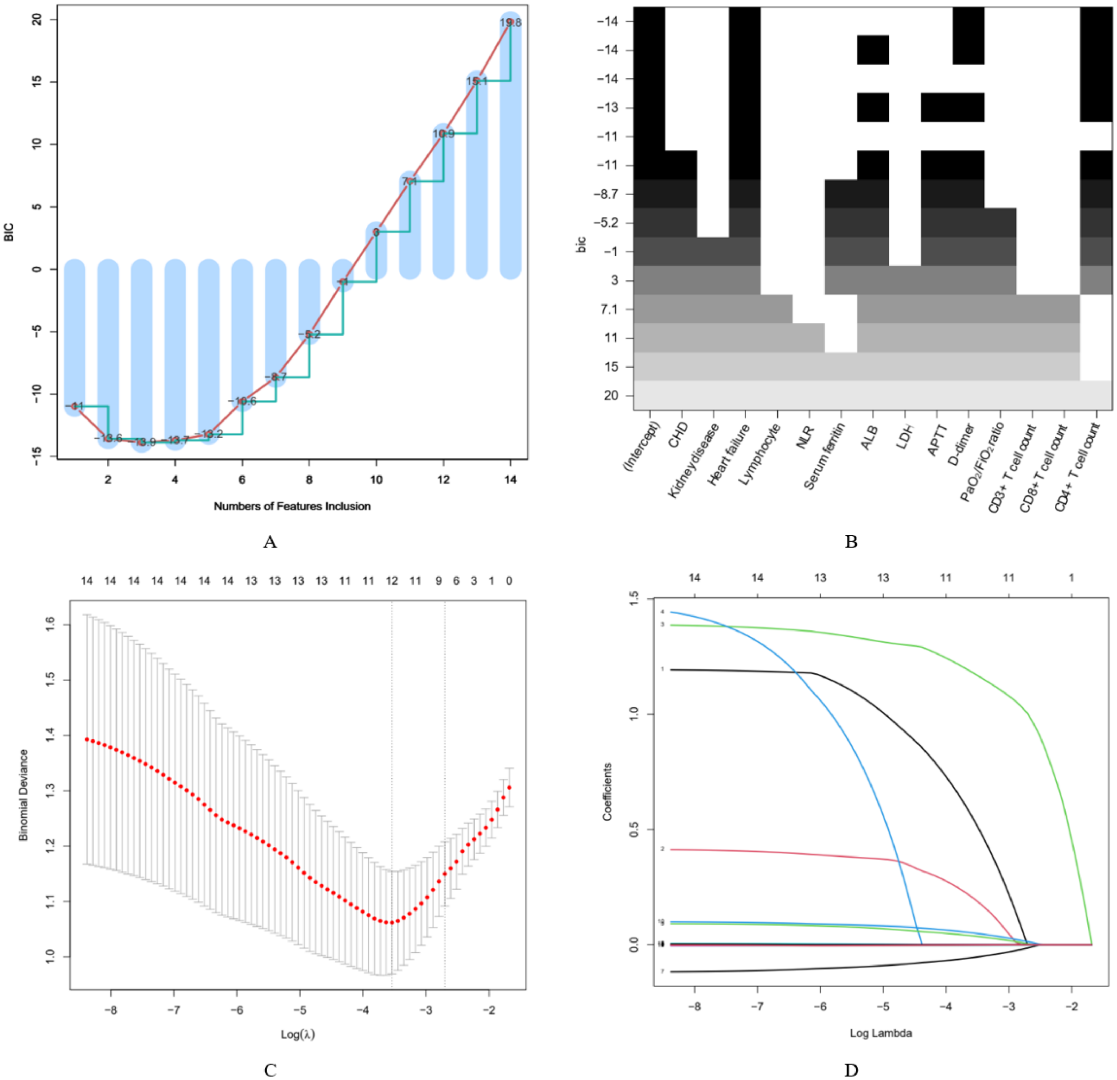
Variable selection. **(A,B)** Variable selection based on minimum Bayesian information criterion (BIC). **(C,D)** Variable selection based on least absolute shrinkage and selection operator (LASSO) regression.

Variables with *p* < 0.10 in univariate analysis (excluding post-admission treatment modalities) were included in the subsequent variable selection process. The optimal subset selection method was employed to determine the best combination of predictive variables by minimizing the Bayesian Information Criterion (BIC). Based on the minimum BIC value, the following variables were selected: heart failure, CD3^+^CD4^+^ T-cell count, and D-dimer (Figures 2A,B).

LASSO regression was employed for variable selection. The model was fitted with family = binomial, and all continuous variables were standardized prior to fitting. The optimal tuning parameter *λ* was selected via 5-fold cross-validation based on the minimization of binomial deviance. According to the one-standard-error rule, λ.1se = 0.067 was chosen as the optimal parameter. At this λ value, six variables with non-zero coefficients were retained, including heart failure (standardized coefficient = 1.004), CD3^+^CD4^+^ T-cell count (standardized coefficient = −0.001), D-dimer (standardized coefficient = 0.014), albumin (standardized coefficient = −0.014), APTT (standardized coefficient = 0.004), and PaO₂/FiO₂ ratio (standardized coefficient = −0.002) (Figures 2C,D).

The variables commonly selected by both methods were heart failure, CD3^+^CD4^+^ T-cell count, and D-dimer. To assess multicollinearity among these three variables, the variance inflation factor (VIF) was calculated. The VIF values were 1.004 for heart failure, 1.004 for CD3^+^CD4^+^ T-cell count, and 1.006 for D-dimer, indicating no significant multicollinearity among the three variables.

Given the established prognostic importance of the SOFA score in clinical mortality risk assessment, it was incorporated alongside the aforementioned three predictors into the final predictive model (Model 2). To enhance clinical interpretability, odds ratios (ORs) for continuous variables were reported per clinically relevant unit change: per 100 cells/μL increase in CD3^+^CD4^+^ T-cell count, per 1 μg/mL increase in D-dimer, and per 1-point increase in SOFA score. Multivariable logistic regression analysis revealed that heart failure was an independent risk factor for in-hospital mortality (adjusted OR = 5.207, 95% CI: 2.027–13.377, *p* = 0.001), whereas a higher CD3^+^CD4^+^ T-cell count emerged as an independent protective factor, with each 100 cells/μL increase associated with an approximate 28.1% reduction in mortality risk (adjusted OR = 0.719, 95% CI: 0.541–0.956, *p* = 0.023). Although D-dimer (adjusted OR = 1.113, *p* = 0.176) and SOFA score (adjusted OR = 1.126, *p* = 0.088) showed trends toward increased risk, neither reached statistical significance (Table 3).

**Table 3:** Multivariable logistic regression model for predicting in-hospital mortality.

Predictor	Adjusted OR (95% CI)	*p*-value
Heart failure (Yes vs. No)	5.207 (2.027–13.377)	0.001
CD3 + CD4 + T-cell count (per 100 cells/μL)	0.719 (0.541–0.956)	0.023
D-dimer, (per 1 μg/mL)	1.113 (0.953–1.300)	0.176
SOFA score (per 1 point)	1.126 (0.982–1.291)	0.088

OR, odds ratio; CI, confidence interval; SOFA, Sequential Organ Failure Assessment.

Continuous variables were scaled as follows: CD3^+^CD4^+^ T-cell count per 100 cells/μL, D-dimer per 1 μg/mL, and SOFA score per 1 point. The OR for CD3^+^CD4^+^ T-cell count was 0.719, indicating that each increase of 100 cells/μL was associated with an approximately 28.1% lower risk of in-hospital mortality.

**Figure 3:**
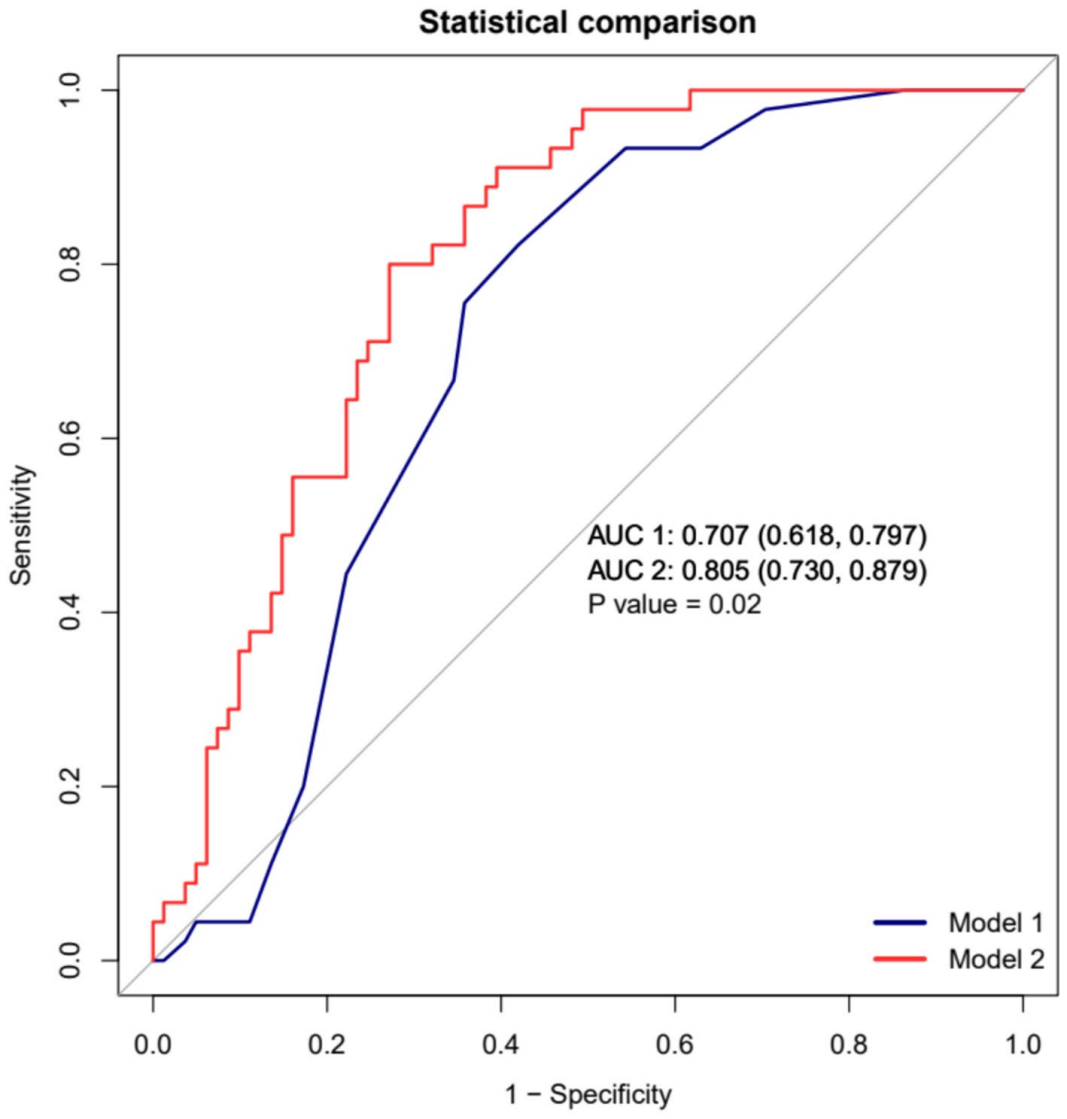
Comparison of receiver operating characteristic curves between the baseline model (Model 1) and the combined clinical predictor model (Model 2) for in-hospital mortality prediction.

Model performance was evaluated by comparing the baseline model (Model 1, SOFA score only) against the combined clinical predictor model (Model 2). Model 2 demonstrated superior discriminative ability, with an area under the receiver operating characteristic curve (AUC) of 0.805 (95% CI: 0.730–0.879), which was significantly higher than that of Model 1 (0.707; 95% CI: 0.618–0.797; *p* = 0.020). At the optimal cut-off value of 0.400, Model 2 achieved a sensitivity of 80.0%, a specificity of 72.8%, and a Youden’s index of 0.528 (Table 4, Figure 3).

**Table 4:** Performance comparison between the baseline model and the combined clinical predictor model.

Model	AUC (95% CI)	Optimal Cut-off	Sensitivity, %	Specificity, %	PPV, %	NPV, %	Youden index
Model 1	0.707 (0.618–0.797)	0.302	82.2	58.0	52.1	85.5	0.403
Model 2	0.805 (0.730–0.879)	0.400	80.0	72.8	62.1	86.8	0.528
*P*-value	0.020						

AUC, area under the receiver operating characteristic curve; CI, confidence interval; PPV, positive predictive value; NPV, negative predictive value. The *p*-value compares the AUCs of the two models (bootstrap test, *n* = 500).

**Figure 4:**
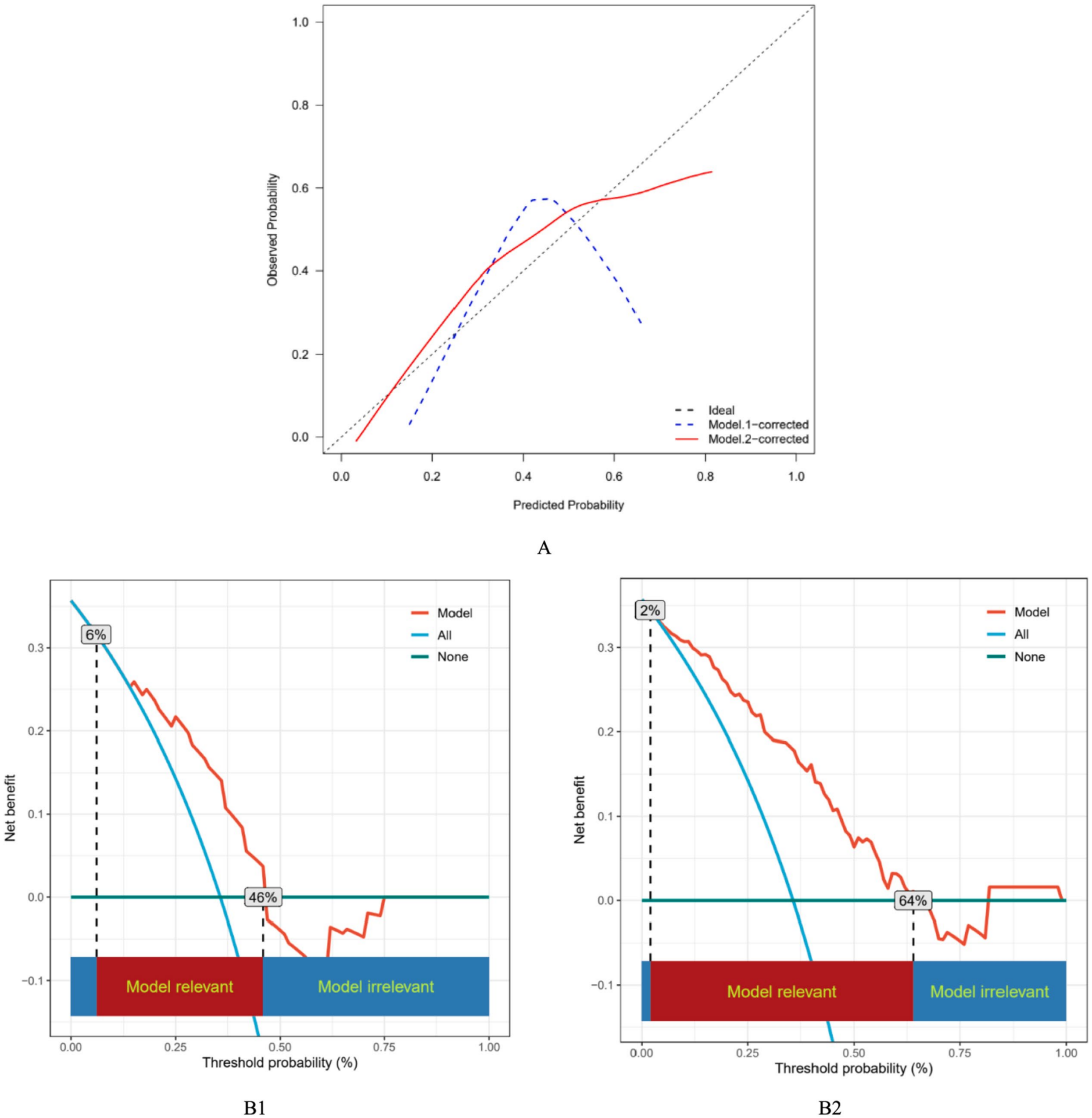
Calibration and decision curve analysis of models for predicting in-hospital mortality risk. **(A)** Calibration curves. The diagonal line represents ideal calibration. The blue dashed line represents Model 1, and the red solid line represents Model 2. **(B)** Decision curve analysis. The blue line (“All”) represents the strategy of intervening for all patients; the green line (“None”) represents intervening for no patients. The red lines denote the net clinical benefit of making intervention decisions based on the risk predictions from the models across various threshold probabilities. B1, Model 1; B2, Model 2.

Calibration analysis demonstrated that Model 2 outperformed Model 1 (Figure 4A). The Brier score of Model 2 was 0.173, lower than that of Model 1 (0.210). The Hosmer-Lemeshow test confirmed good calibration for Model 2 (*χ*^2^ = 5.426, *p* = 0.711), whereas it could not be reliably calculated for Model 1 due to sparse expected frequencies. These findings indicate that the combined model provides better predictive calibration.

Decision curve analysis was used to assess the clinical utility of the models. Model 1 had a clinically effective threshold range of 6–46% and a peak net benefit of 0.32. Model 2 had a clinically effective threshold range of 2–64% and a peak net benefit of 0.35. Within the entire overlapping threshold range, the net benefit curve of Model 2 was consistently above that of Model 1. In addition, both models achieved significantly greater net benefit than the “treat none” and “treat all” strategies (Figure 4B).

### 3.4 Internal validation of the multivariable model

Internal validation of the combined prediction model (Model 2) was performed using bootstrap resampling with 500 iterations. The optimism-corrected C-statistic (AUC) was 0.788, with a Nagelkerke *R*^2^ of 0.301. Calibration assessment demonstrated an optimism-corrected intercept of −0.036 and slope of 0.899, indicating good calibration. The corrected Brier score was 0.186 (Table 5).

**Table 5:** Internal validation of the prediction model.

Performance metric	Apparent (Original)	Optimism-Corrected
Discrimination		
C-statistic (AUC)	0.805	0.788
Nagelkerke’s *R*^2^	0.349	0.301
Calibration		
Intercept	0	−0.036
Slope	1	0.899
Overall performance		
Brier score	0.173	0.186

Based on 500 bootstrap resamples. AUC, area under the receiver operating characteristic curve.

## 4 Discussion

In this single-center retrospective study of adult patients with p-ARDS, we evaluated the associations between underlying diseases, demographic characteristics, laboratory parameters within 48 h of admission, and in-hospital mortality risk. Based on these findings, we developed and internally validated a multivariable prediction model (Model 2) incorporating a history of heart failure, CD3 + CD4 + T cell count, D-dimer, and SOFA score. Compared with the baseline SOFA-only model (Model 1), Model 2 showed better discriminative ability, improved calibration, and favorable clinical utility, providing a more precise tool for early risk stratification in this high-risk population.

Our study found that a decrease in CD3 + CD4 + T cell count is an independent risk factor for in-hospital mortality in patients with p-ARDS. Following pathogen invasion, complex negative feedback and exhaustion of the immune system can lead the host into a state of immunoparalysis (15), a complex immune dysregulation that renders the host unable to effectively clear pathogens while continuously suffering from inflammatory damage. CD4 + T-cell depletion in critically ill patients involves complex interactions between cytokine signaling and transcriptional reprogramming. Chronic immune activation causes T-cell exhaustion, characterized by effector function loss, inhibitory receptor upregulation, and reduced proliferation (16). A recent single-cell multiomic study identified an NR4A2^+^ exhausted central memory CD4 + T-cell subset in abdominal, pulmonary, and skin-derived sepsis. Genetic perturbation experiments showed that Nr4a2 knockout improved survival in septic mice, while overexpression worsened outcomes (17). In sepsis, CD4 + T-cell impairment is multipronged. Beyond acute-phase cell loss, survivors often develop persistent immune suppression driven by lymphopenia-induced homeostatic proliferation. This leads to increased nosocomial infection susceptibility, altered phenotype/function, reduced TCR diversity, and changes in CD4 + subpopulations (18). Our findings provide clinical evidence for the immunopathological mechanism of infection-related ARDS. Previous studies have confirmed that severe infections can trigger “immune exhaustion” or “immunoparalysis” in the host, characterized in COVID-19 by lymphopenia and functional suppression, primarily of CD3 + CD4 + T cell (19, 20). CD3 + CD4 + T cell was crucial for coordinating anti-infection immunity, and their reduction is closely associated with an increased risk of secondary infections, delayed viral clearance, and impaired tissue repair (21). Our findings are consistent with existing evidence, suggesting that peripheral blood lymphocyte subsets may serve as effective biomarkers for assessing immune status and predicting prognosis in critically ill patients, thereby complementing the limitations of traditional tools such as the SOFA score and CURB-65 score in reflecting host immune status. However, our study is a preliminary, exploratory investigation based on a small sample of 126 patients. Larger prospective studies are warranted to validate our findings.

NLR reflects the complex interplay between innate and adaptive immunity-a key determinant of severity in pneumonia-related ARDS. Cataudella et al. (22) showed in 195 older patients with community-acquired pneumonia that NLR predicted 30-day mortality more accurately than PSI, CURB-65, CRP, and white blood cell count. Regolo et al. (23) reported that NLR independently predicted mortality and worse outcomes in COVID-19 patients. A subsequent study by the same group found that the effect of inflammation on the P/F ratio was partially mediated by neutrophils (24). In our study, univariate analysis showed a borderline association between NLR and mortality (OR = 1.013, *p* = 0.051), but NLR was not retained in the final multivariable model after BIC and LASSO selection. This discrepancy may be explained by our limited sample size. Nevertheless, given its low cost and biological rationale, NLR remains an attractive adjunctive biomarker. Larger prospective studies are needed to clarify its independent prognostic role in multidimensional prediction models for pneumonia-related ARDS.

Inflammation and coagulation, as core physiological responses of the host to infection and injury, play a crucial role in the pathogenesis of ARDS (25). Studies have confirmed that thrombosis and coagulation dysfunction are important pathological bases for the development of ARDS (26). Endotoxins or bacteria can activate tissue factor, and alveolar epithelial cells release tissue factor-bearing microparticles, thereby initiating the coagulation cascade and promoting thrombin generation and widespread fibrin deposition (27). In this study, D-dimer was ultimately retained following a rigorous variable selection strategy. Although D-dimer did not reach the conventional level of statistical significance in the final multivariable logistic regression model, its selection by two distinct methodological approaches provides stronger evidence of its predictive robustness than a single *p*-value alone. The final model incorporating D-dimer showed good discrimination (AUC = 0.805) and calibration (Hosmer-Lemeshow test *p* = 0.711). Bootstrap internal validation (500 resamples) demonstrated minimal optimism (optimism-corrected AUC = 0.788), indicating a low risk of overfitting. These findings collectively support that D-dimer contributes meaningfully to the overall predictive performance of the model. Previous studies have confirmed that elevated D-dimer levels are closely associated with poor prognosis in patients with ARDS, particularly in COVID-19-related ARDS, and show a positive correlation with disease severity (28). Our findings further support D-dimer as a potential biomarker for early risk stratification in patients with pneumonia-associated ARDS (p-ARDS).

This study showed that a history of heart failure remained a strong independent predictor of mortality in p-ARDS patients, even after adjusting for the SOFA score. Although previous definitions of ARDS excluded volume overload or heart failure, recent evidence suggests that these conditions may coexist with ARDS in up to one-third of patients (4). Patients with heart failure have insufficient cardiac pump reserve and chronic hypoperfusion. When ARDS occurs, hypoxemia and mechanical ventilation may further increase cardiac preload and afterload. Concurrently, systemic inflammation and infection can directly impair myocardial function, thereby compromising the patient’s compensatory capacity during acute illness. These findings highlight the importance of systematic evaluation and active management of underlying cardiac function in patients with p-ARDS.

Nevertheless, several limitations of this study should be acknowledged. First, the EPV for our model (four predictors) was 11.25 (based on 45 deaths), exceeding the recommended threshold of 10. Bootstrap internal validation (500 resamples) showed minimal optimism (corrected AUC = 0.788), indicating a low risk of overfitting. However, the lack of an external validation dataset is a major limitation, and our findings require further validation in prospective cohorts. We will continue to accumulate later cohort data from our center to conduct external validation studies. Second, the retrospective design may be subject to limitations regarding the completeness and accuracy of data documentation. The main predictor of interest in this study, lymphocyte subsets, was not a routine admission test but was selectively ordered by clinicians based on their assessment of disease severity. Consequently, a total of 1,282 patients from the same time period were excluded due to missing this data, leaving only 126 patients in the final analysis. The missing mechanism for this variable is likely missing not at random (MNAR). Under this circumstance, any imputation method could potentially introduce unreliable or even misleading estimates. Therefore, we adopted complete case analysis as the most honest and conservative strategy given the current dataset. We acknowledge that excluding such a high proportion of patients indeed limits the generalizability of our findings. Furthermore, due to the inherent constraints of missing data, we were unable to perform conventional sensitivity analyses such as multiple imputation. Future external validation in independent cohorts is needed to further assess the generalizability of our model. Third, this study only measured baseline immune markers within 48 h of admission without dynamic monitoring. A single time-point measurement may not fully reflect dynamic changes in immune status and may underestimate the prognostic value of immune markers. Future studies should include monitoring at multiple time points (e.g., day 3, day 7) to investigate the relationship between immune marker trajectories and prognosis. Fourth, due to the limitation of the study population to immunocompetent adults with p-ARDS, the findings should be regarded as exploratory. The results may not be generalizable to ARDS of other etiologies (e.g., trauma or pancreatitis), and thus, caution should be exercised when extrapolating the conclusions to ARDS arising from different causes. Fifth, the study was designed for clinical prediction model development rather than mechanistic exploration. The proposed mechanistic explanations should be considered speculative. Future prospective studies incorporating multi-omics approaches (e.g., transcriptomics, proteomics, epigenomics) alongside comprehensive clinical phenotyping are needed to elucidate the underlying biological pathways. Sixth, the data-driven variable selection strategy employed in this study may have omitted certain clinically important variables. For example, age and some comorbidities were not included in the model because they showed a weak association with mortality in this dataset (univariate *p* > 0.10) or were not selected by both methods simultaneously. Given the limited sample size, this strategy helped ensure model robustness and reduce overfitting. However, we acknowledge that some clinical variables clearly associated with ARDS mortality may still have been excluded. Future external validation in larger, independent cohorts is needed.

In summary, we have successfully developed and validated an in-hospital mortality prediction model for adult patients with p-ARDS. This model integrates SOFA score, underlying comorbidities (heart failure), immune status (CD3 + CD4 + T cell count), and coagulation function (D-dimer), effectively complementing and improving traditional scoring systems. As a preliminary exploratory study, future efforts will focus on external validation in prospective multicenter cohorts and the addition of dynamically monitored biomarkers to further improve predictive accuracy.

## Data Availability

The original contributions presented in the study are included in the article/supplementary material, further inquiries can be directed to the corresponding authors.
